# Severe Pandemic H1N1 2009 Infection Is Associated with Transient NK and T Deficiency and Aberrant CD8 Responses

**DOI:** 10.1371/journal.pone.0031535

**Published:** 2012-02-20

**Authors:** Annette Fox, Le Nguyen Minh Hoa, Peter Horby, H. Rogier van Doorn, Nguyen Vu Trung, Nguyen Hong Ha, Nguyen Trung Cap, Vu Dinh Phu, Nguyen Minh Ha, Diep Nguyen Thi Ngoc, Bich Vu Thi Ngoc, Huong Tran Thi Kieu, Walter R. Taylor, Jeremy Farrar, Heiman Wertheim, Nguyen Van Kinh

**Affiliations:** 1 Oxford University Clinical Research Unit, Wellcome Trust Major Overseas Programme, Dong Da, Ha Noi, Viet Nam; 2 Nuffield Department of Clinical Medicine, Centre for Tropical Medicine, University of Oxford, Oxford, United Kingdom; 3 National Hospital for Tropical Diseases, Dong Da, Ha Noi, Viet Nam; 4 South East Asia Infectious Diseases Clinical Research Network, Jakarta, Indonesia; Statens Serum Institute, Denmark

## Abstract

**Background:**

It is unclear why the severity of influenza varies in healthy adults or why the burden of severe influenza shifts to young adults when pandemic strains emerge. One possibility is that cross-protective T cell responses wane in this age group in the absence of recent infection. We therefore compared the acute cellular immune response in previously healthy adults with severe versus mild pandemic H1N1 infection.

**Methods and Principal Findings:**

49 previously healthy adults admitted to the National Hospital of Tropical Diseases, Viet Nam with RT-PCR-confirmed 2009 H1N1 infection were prospectively enrolled. 39 recovered quickly whereas 10 developed severe symptoms requiring supplemental oxygen and prolonged hospitalization. Peripheral blood lymphocyte subset counts and activation (HLADR, CD38) and differentiation (CD27, CD28) marker expression were determined on days 0, 2, 5, 10, 14 and 28 by flow cytometry. NK, CD4 and CD8 lymphopenia developed in 100%, 90% and 60% of severe cases versus 13% (p<0.001), 28%, (p = 0.001) and 18% (p = 0.014) of mild cases. CD4 and NK counts normalized following recovery. B cell counts were not significantly associated with severity. CD8 activation peaked 6–8 days after mild influenza onset, when 13% (6–22%) were HLADR+CD38+, and was accompanied by a significant loss of resting/CD27+CD28+ cells without accumulation of CD27+CD28− or CD27−CD28− cells. In severe influenza CD8 activation peaked more than 9 days post-onset, and/or was excessive (30–90% HLADR+CD38+) in association with accumulation of CD27+CD28− cells and maintenance of CD8 counts.

**Conclusion:**

Severe influenza is associated with transient T and NK cell deficiency. CD8 phenotype changes during mild influenza are consistent with a rapidly resolving memory response whereas in severe influenza activation is either delayed or excessive, and partially differentiated cells accumulate within blood indicating that recruitment of effector cells to the lung could be impaired.

## Introduction

In March 2009 a novel influenza A virus (A/California/04/2009(H1N1): 2009 H1N1) was introduced into the human population and then spread globally. It was first detected in Viet Nam in Ho Chi Minh City in May 2009 and in Ha Noi in June [1]. This 2009 H1N1 virus contained a unique combination of gene segments from North American classical swine H1N1, Eurasian swine H1N1 and triple-reassortant swine H1N2 lineages [2] including antigenically novel haemagglutinin (HA) [2] and neuraminidase (NA) proteins [3]. Accordingly, the very small proportion of people that had detectable neutralizing or NA-inhibiting antibodies prior to the pandemic were elderly and likely to have been infected with H1N1 viruses closely related to those circulating between 1918 and 1957 [3]–[6].

The medically attended case fatality rate was less than 0.05% during the first wave of the pandemic, which is low compared to previous pandemics [7]–[9]. However, up to a third of severe and fatal cases were previously healthy young to middle-aged persons, a group that is generally spared during seasonal epidemics, which predominantly affect the very young because they are immunologically naïve and the elderly because of immune-senescence [7], [10]–[11]. A similar but stronger trend was seen during the 1918 pandemic when mortality was high in the very young, adults aged 20–40 years and the elderly with relative sparing of children and older adults, resulting in a W-shaped mortality curve [12]. It's unclear why outcomes vary in healthy adults or why the burden of severe influenza sometimes shifts to young adults when pandemic strains emerge. A number of potential and possibly interacting explanations have been proposed. These include: exposure of older adults to similar stains in past decades, i.e. influenza recycling [13]; age-related differences in bacterial carriage and superinfection [13]; putative age-related differences in immune regulation that render children less susceptible to immune pathology [14], and protection by cross-reactive immune responses induced by prior seasonal influenza exposure [15]. Given that most people have had influenza by the age of 6 [16], an extension of the latter theory must be that cross-reactive immune responses wane.

CD8 T cells are important mediators of cross-reactive clinical influenza immunity in animal models whereby memory T cells recognize conserved viral proteins and limit virus growth such that viral loads decline more rapidly and clinical symptoms are reduced [17]–[21]. Human CD8 T cells kill influenza A virus infected cells *in vitro* and are associated with faster clearance of an antigenically distinct attenuated virus strain *in vivo* [22] but their contribution to clinical protection is debated. It has been suggested that cross-reactive T cells contribute little to clinical protection against pandemic viruses since morbidity and mortality were high in 1918, 1957, and 1968 when H1N1, H2N2 and H3N2 emerged [23]. However, adults with recent seasonal influenza were less likely to develop influenza during the 1957 [24] and 1968 pandemics [25] suggesting that cross-protection exists, although immune responses were not compared to verify this. Limited data also indicate that influenza-specific CD8 cytotoxic T cell activity declines sharply after infection [26] such that age groups with low seasonal influenza infection rates may be more susceptible to clinical illness during pandemics. The few studies that have used serology to identify asymptomatic as well as symptomatic seasonal influenza infections in the community find that infection rates decline with age or show a wave like pattern, with rates being lowest in young adults [27]–[28]. We have also observed this wave-like pattern in a Vietnamese community followed since 2007 (Horby et al in preparation).

To investigate whether the occurrence of severe 2009 H1N1 pandemic influenza infection in previously healthy adults is associated with cellular immune responses we examined the absolute number, and activation and differentiation status of peripheral blood lymphocyte subsets over the course of severe versus mild illness. We focus on CD8 T cells because they can recognize the highly conserved internal influenza proteins and thereby have greater potential to recognize pandemic 2009 H1N1. HLA-DR and CD38 co-staining was used to estimate the percentage responding to influenza since the vast majority of CD8 T cells co-expressing these markers are specific for the infecting agent [29]. CD28 and CD27 co-staining was used to assess CD8 T cell differentiation as the successive loss of these co-stimulatory molecules following activation marks the acquisition of proliferative capacity followed by cytotoxic/antiviral function [30]–[31].

## Results

### Clinical characteristics of patients classified as severe versus mild influenza

Between 28 September 2009 and 23 January 2010 231 patients with suspected influenza were screened, 114 (49%) had virologically confirmed 2009 H1N1 infection, and 62 (54%) were enrolled. Thirteen patients were excluded from analysis in this study, 12 because they had pre-existing systemic or respiratory conditions and one because they were aged <15 years (Figure S1). The pre-existing conditions were pregnancy (2), leukemia (3), laryngopharynx cancer (1), hypertension (1), congenital heart disease (1), chronic sinusitis (3), and asthma (1). None of the patients were obese, defined as body mass index (BMI) ≥30 according to BMI classification of the WHO Global Database on BMI. Forty nine (79%) of the enrolled patients were previously healthy adults of which 10 had severe illness and 39 mild illness. Two of the patients with severe illness died, both were females, one aged 23 and the other 42 years. Severe and mild patients were not significantly different in terms of age or sex (Table 1). The number of days from onset to admission and spent in hospital were both significantly greater for severely ill patients (Table 1). All patients with mild illness had recovered and were discharged within 2 weeks of onset whereas 5 of the 8 surviving severe patients were still admitted at this time. Tachypnea, tachycardia and hypoxemia were significantly more common in patients with severe illness, both at enrolment and during admission, consistent with the inclusion criteria (Table 1). Patients with severe illness received oxygen for a median of 4 days (1–11 days) and 3 were mechanically ventilated. The adapted PMEWS, a clinical severity score, was higher for severely ill patients (Table 1).

**Table 1:** Patient characteristics.

	Mild	Severe	p
	**Median (10–90% range)**		
Age	27.4 (19.2–37.1)	36.5 (20.2–56.3)	0.142
BMI	20.1 (16.8–25.3)	19.1 (18.0–20.4)	0.608
Days Ill at Admission	2 (1–4)	3(2–6)	0.003
Days Ill at Enrollment	3 (2–4)	5.5 (3.1–9)	<0.001
Days hospitalized	7 (5–12)	14 (8–17)	0.001
adapted PMEWS*	3 (2–6)	9 (6–18)	<0.001
	**n/N (%)**		
Male	15/39 (38.5)	5/10 (50.0)	0.720
**Status at enrolment**			
Temperature ≥38°C	12/39 (30.8)	6/10 (60.0)	0.141
Tachypnea (rpm>30)	0/39 (0.0)	5/10 (50.0)	<0.001
Hypoxemia (SpO2 < = 92)	0/39 (0.0)	3/10 (30.0)	0.007
Tachycardia (bpm>100)	1/38 (2.6)	5/10 (50.0)	0.001
**Status during admission**			
Tachypnea	0/39 (0.0)	6/10 (60.0)	<0.001
Hypoxemia	1/39 (2.6)	6/10 (60.0)	<0.001
Tachycardia	3/39 (7.7)	8/10 (80.0)	<0.001

*adapted PMEWS = adapted pandemic medical early warning score as defined in the methods.

**Figure 1:**
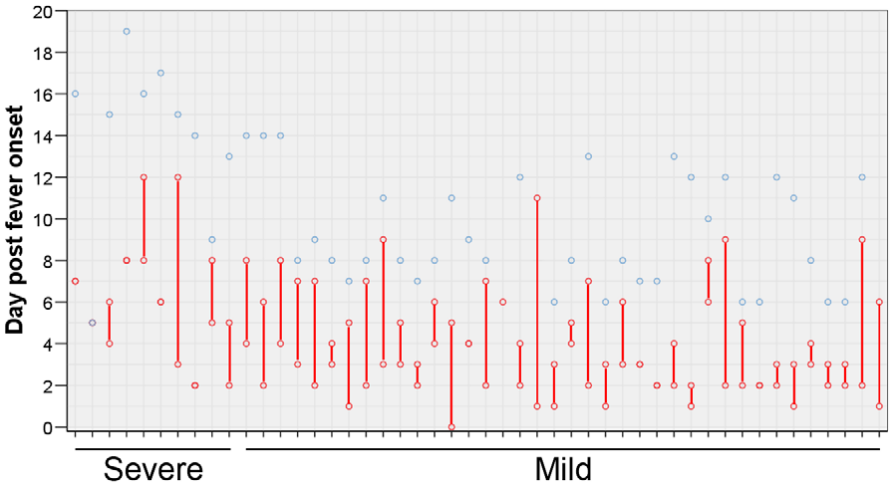
Detection of influenza virus RNA in respiratory specimens from patients with severe versus mild influenza. Nose and throat swabs were assessed for the presence of viral RNA as described in the methods. Red dots/lines indicate the first and last days from fever onset when viral RNA was detected, the first day being when the first sample was collected. Blue dots represent the last day that samples were tested.

The median day of illness when viral RNA was last detected in nose and/or throat swabs was 5 (3–9) with no significant difference between severe and mild cases (Figure 1, p = 0.102). There were insufficient samples with detectable viral loads to investigate the relationship with severity; however we could not detect any significant difference in Ct values for CDC influenza A realtime RT-PCR with respiratory samples from severe versus mild patients (data not shown). Only one patient had influenza virus RNA detected in plasma, and this was a patient that died (data not shown).

### Absolute lymphocyte subset counts in severe versus mild influenza

**Figure 2:**
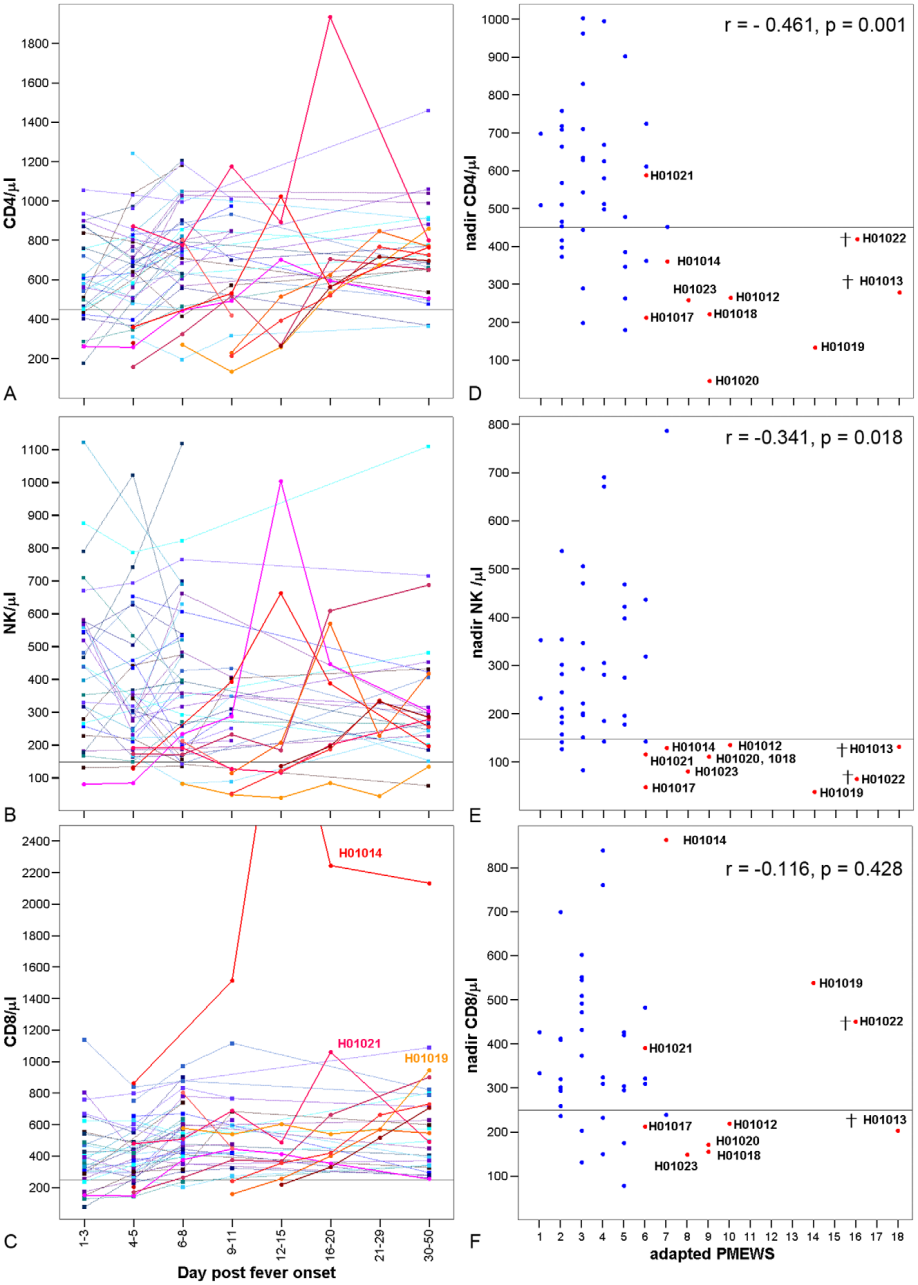
T and NK cell counts in severe versus mild influenza. Panel's a–c show lymphocyte subset counts by time interval since onset with each line representing an individual patient. Horizontal solid lines represent the lower limit of the normal range. Panels d–f show nadir values for lymphocyte subset counts versus pandemic medical early warning score (PMEWS) with Spearman's correlation coefficients. The 2 patients that died are indicated ( ) because they were only assessed at one or two time points. Results are shown for 10 patients with severe illness (red tone symbols and lines) and 39 patients with mild illness (blue tone symbols and lines).

Lymphopenia was significantly more common in patients with severe illness at enrolment and during admission, but not at follow-up (Table 2). Lymphopenia was mainly due to low CD4 and NK cell counts, which fell below the normal range in 90% and 100% of severe patients, respectively, reflecting significantly lower nadir levels than in mild patients (Table 2, Figure 2 a&b). CD4 and NK counts were also significantly associated with the adapted PMEWS (Figure 2d&e). CD4 (Figure S2) but not NK counts (data not shown) were associated with the cycle threshold (Ct) value in the CDC influenza A RT-PCR, a semi-quantitative indicator of the amount of viral RNA in swabs. CD8 lymphopenia was detected in 6 (60%) of the severe patients compared to 7 (18%) of the mild patients (Table 2) but CD8 counts were normal or high over the course of acute illness in the remaining 4 severe patients (Figure 1c) such that median counts did not differ significantly between severe and mild patients (Table 2). CD4∶CD8 ratios were significantly lower in severely ill patients at enrolment (p = 0.016, data not shown) and during admission (Table 2), when 70% of severe versus 16% of mild patients had ratios less than 1 (p = 0.002). B cell lymphopenia was detected in only 2 severe patients, of which 1 had B cell lymphopenia following recovery, and B cell counts did not differ significantly between severe and mild patients (Table 2).

**Table 2:** Analysis of lymphocytes and subsets.

	Mild	Severe	p
**Status at Enrolment** *	**n/N (%)**		
Lymphopenia	10/39 (26)	6/9 (67)	0.044
CD3 T lymphopenia	12/39 (31)	5/9 (56)	0.247
CD4 T lymphopenia	9/39 (23)	7/9 (78)	0.004
CD8 T lymphopenia	6/39 (15)	5/9 (56)	0.020
NK lymphopenia	1/39 (3)	6/9 (67)	<0.001
CD19 B lymphopenia	1/39 (3)	0/9 (0)	1.000
**Status while admitted**			
Lymphopenia	12/39 (31)	8/10 (80)	0.009
CD3 lymphopenia	15/39 (38)	7/10 (70)	0.090
CD4 lymphopenia	11/39 (28)	9/10 (90)	0.001
CD8 lymphopenia	7/39 (18)	6/10 (60)	0.014
NK lymphopenia	5/39 (13)	10/10 (100)	<0.001
CD19(B) lymphopenia	3/39 (8)	2/10 (20)	0.267
**Status at Follow-up** **			
Lymphopenia	2/19 (10)	1/8 (12)	1.000
CD3 T lymphopenia	2/19 (10)	1/8 (12)	1.000
CD4 T lymphopenia	1/19 (5)	0/8 (0)	1.000
CD8 T lymphopenia	0/19 (0)	0/8 (0)	1.000
NK lymphopenia	1/19 (5)	1/8 (12)	0.513
CD19 B lymphopenia	0/19 (0)	1/8 (12)	0.258
**Nadir values**	**Median cells/µl (10–90% range)**		
CD45+/lymphocytes	1625 (983–2155)	930 (577–1527)	<0.001
CD3 T cells	1019 (699–1683)	535 (385–1272)	0.003
CD4 T cells	543 (346–901)	261 (53–570)	<0.001
CD8 T cells	324 (203–602)	215 (149–830)	0.196
NK cells	245 (143–537)	111 (39–135)	<0.001
CD19 B cells	155 (104–240)	97 (48–443)	0.110
CD4∶CD8 ratio	1.32 (0.83–2.06)	0.92 (0.14–1.50)	0.005

*1 severely ill patient was not assessed at enrolment.

**The day post-onset for follow-up assessments was 35 (31–47) for mild patients and 34 (31–37) for severe patients.

Since severely ill patients presented later, lymphocyte counts were compared for severe versus mild patients tested on equivalent days since fever onset (Figure 2 a&b). CD4 counts were significantly lower in severe compared to mild patients on days 4–5 (p = 0.014); 6–8 (p = 0.032) and days 9–11 post-onset (p = 0.027) but not on days 30–50 (p = 0.775). NK cell counts were also significantly lower in severe patients on days 4–5 (p<0.001), 6–8 (p = 0.018) and 9–11 (p = 0.036) but not on days 30–50 post onset (p = 0.775). In patients with mild illness CD4 counts were lower 1–3 days compared to 6–8 days post-onset (p = 0.009), when levels were similar to those following recovery (p = 0.780 )(Figure 2a). Although CD4 counts also increased with time since onset in severe patients, half were still CD4 lymphopenic 9–11 days post-onset.

### CD8 T cell activation and differentiation during mild influenza

**Figure 3:**
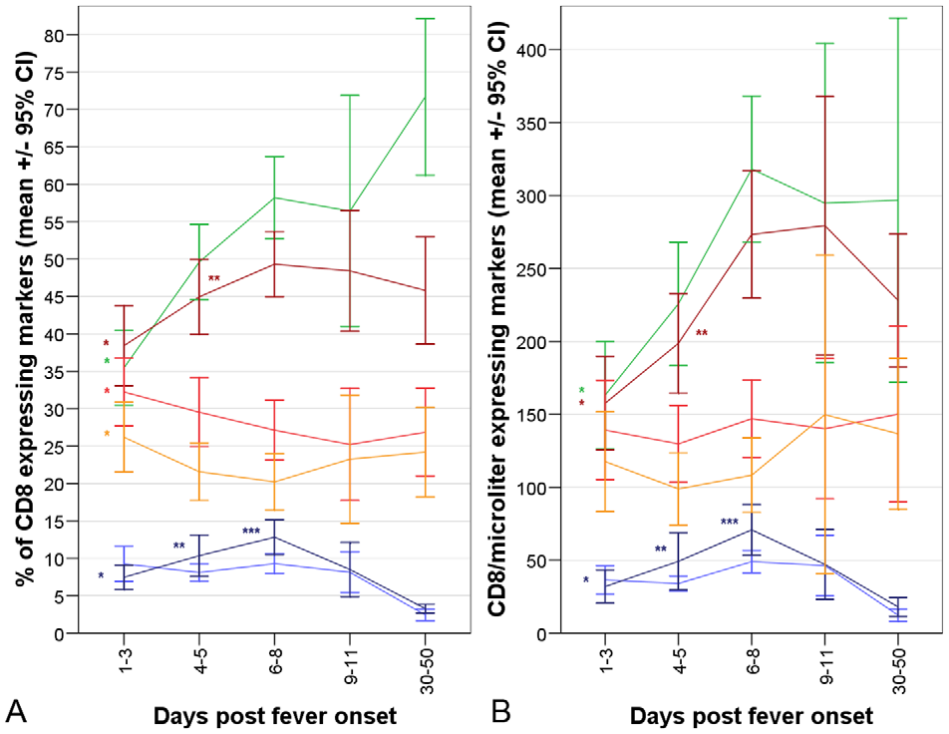
Activation, differentiation and extravasation marker expression by CD8 T cells over the course of mild influenza A illness. The percentages (a) and absolute count (b) of CD8 T cells with the phenotypes CXCR3^high^ (green lines); CD27+CD28+ (brown lines); CD27+CD28−(red lines); CD27−CD28− (yellow lines); CD38+HLA-DR- (light blue lines) and CD38+HLADR+ (dark blue lines) is shown for 39 patients with mild influenza illness at different time intervals post-fever onset. The number tested during each time interval was 28, 34, 39, 19 and 9. Asterisks indicate where values differ significantly via paired t-Test from levels at recovery (*), or at recovery and day 1–3 (**), or at recovery and at earlier time intervals (***).

In patients with mild illness the percentage and absolute count of CD8 T cells expressing activation markers CD38 and HLADR was significantly higher 1–3 days post onset than 30–50 days post-onset, i.e. following recovery (Figure 3, Figure S3). CD38+HLA-DR+ CD8 frequencies peaked 6–8 days post-onset when the mean was 13% (95%CI 6–22%) of CD8 T cells or 70 cells/µl (95%CI 21–127cells/µl) were positive (Figure 3). At the earliest times post-onset a higher percentage of cells expressed CD38 alone than expressed CD38 and HLADR, consistent with this being a marker expressed earlier after activation (Figure 3a). The percentage of CD8 T cells with a resting CD27+CD28+ phenotype was significantly lower at 1–3 days post-onset than at later times and returned to post-recovery levels by 6–8 days post-onset (Figure 3a, Figure S3). In accompaniment the percentages with CD27+CD28− (intermediate differentiation) and CD27−CD28− (late differentiation) phenotypes were highest 1–3 days post onset and returned to post-recovery levels by 6–8 days post-onset (Figure 3a). Absolute counts for CD27+CD28+ CD8 T-cells were also lowest 1–3 days post-onset but CD27+CD28− and CD27−CD28− CD8 T cell counts were not increased (Figure 3b). The percentage and number of CD8 T-cells with a CXCR3^high^phenotype was significantly lower 1–3 days after onset than following recovery and gradually increased.

### CD8 T cell activation and differentiation in severe compared to mild influenza

**Figure 4:**
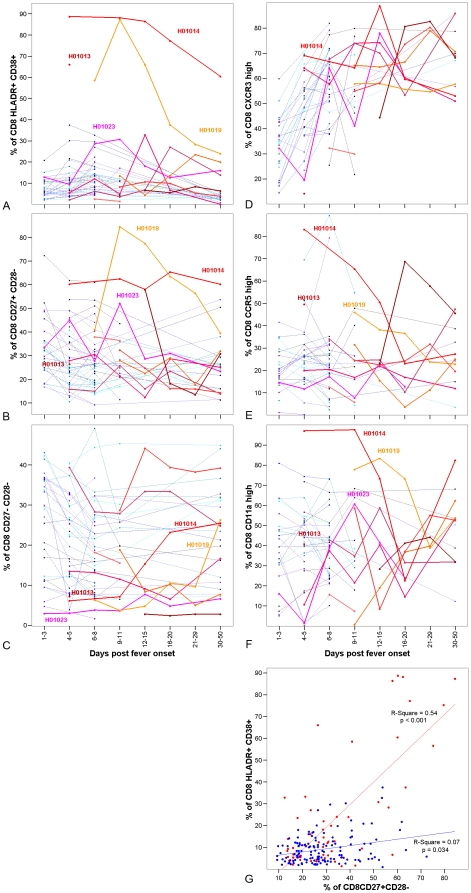
Activation and differentiation marker expression by CD8 T cells from patients with severe versus mild influenza. Panels a–f show the percentages of cells expressing the markers indicated on the vertical axis by time interval since onset. Each line represents an individual patient. Panel g shows the relationship between the percentages of CD8 T cells that are CD38+HLADR+ and CD27+CD28− and includes results for all time points. Results are shown for 10 patients with severe influenza (red tones symbols and lines) and 39 patients with mild influenza (blue tone symbols and lines).

The peak percentage of CD8 T cells expressing CD38 and HLADR activation markers was higher in severe influenza (30%, (4–89%), p = 0.019), but varied widely in terms of timing and magnitude (Figure 4a, Figure S3). In 4 severe patients more than 30% of CD8 T cells were CD38+HLADR+ by day 8 of illness. In the remainder the percentage CD38+HLADR+ was low or increased more than 9 days after onset whereas activated CD8 T-cell frequencies were declining by this time in mild illness. The caveat being that 3 severe patients presented more than 8 days after onset such that an earlier activation response may have been missed. Differentiation and extravasation marker expression also varied markedly (Figure 4 b&c) and was significantly associated with activation marker expression in severe influenza (Figure 4g). CD27+CD28−/partially differentiated (Figure 4b), CDlla^high^ (Figure 4e) and CCR5^high^ (Figure 4f) cells predominated amongst CD8 T cells from patients with excessive activation. Consequently, the percentage of CD8 T cells with a CD27−CD28−/fully differentiated phenotype was low in these patients (Figure 4c).

## Discussion

In this study we demonstrate that acute immune responses following 2009 H1N1 infection differ between previously healthy young adults that develop severe versus mild illness. Lymphopenia was significantly more common in severe illness and CD4 and NK cells were the major subsets contributing to lymphopenia, with no detectable B cell involvement. CD8 lymphopenia was detected in fewer severe patients but was still more common than in patients with mild illness. The CD8 T cell activation and differentiation response also differed, being rapid, transient and modest in patients with mild illness but delayed and/or excessive in patients with severe illness.

Mechanisms contributing to lymphopenia include reduced lymphocyte production, increased apoptosis or necrosis, and/or increased lymphocyte marginalization or trapping. As will be discussed, all of these mechanisms have been implicated in lymphopenia during severe influenza infection but the predisposing factors have not been fully elucidated and it is not know if lymphopenia contributes to severity or is a consequence. Lymphopenia has been associated with the severity of infection with influenza A viruses that vary in intrinsic virulence including pandemic H1N1 [10], [32]–[33], seasonal A/H3N2 [34], and A/H5N1 [35]–[37]. Lymphopenia was also observed in nearly one third of mild influenza cases but was relatively transient, consistent with reports elsewhere [1], [38]–[40], and probably reflects transient loss of T cells from the blood as they migrate to draining lymph nodes [19]. This process is unlikely to adequately account for the severe and prolonged lymphopenia observed in severely ill patients. A substantial proportion of severe patients remained lymphopenic 9–11 days post onset, similar to reports for severe pandemic H1N1 patients from China [33]. In the current study lymphocyte subset counts were also performed, which clearly show that lymphopenia is predominantly attributable to CD4 and NK cell depletion. Several other studies also find that lymphopenia is associated with CD4 T cell depletion in severe and/or mild influenza [33], [41]–[43]. Consequently inverted CD4∶CD8 ratios were more common in severe patients. In 60% of severe patients CD8 T-cell counts also fell below the normal range whereas the remainder had normal or high CD8 counts as well as excessive CD8 T-cell activation. This indicates that the mechanism underlying lymphopenia may affect both T cell subsets but losses in the CD8 subset may be offset by activation and proliferation. Lymphopenia in mice infected with highly pathogenic H5N1 is associated with increased in situ detection of apoptotic cells in lung and lymphoid tissue [44] and lymphoid atrophy has been observed in several severe 2009 H1N1 patients [45]. A large proportion of lymphocytes undergo apoptosis following antigen driven activation and expansion [46], and activated CD8 T cell frequencies were high in severe influenza indicating that activation induced apoptosis may contribute to lymphopenia. Moreover, others have demonstrated that activated CD4 T cell frequencies are also higher in severe 2009 H1N1 infection [47] and that a greatly increased and massive proportion of CD4 T cells express CD95 (the death receptor), with a lesser increase in the proportion of CD8 T cells expressing CD95 [41]. The association between activation, apoptosis and peripheral T cell depletion is best described for HIV infection where depletion is also greatest amongst the CD4 subset, a phenomenon attributed to greater expansion potential of CD8 than CD4 T cells following activation [46]. There was a weak association between higher values in semi-quantitative assessment of viral RNA and lower CD4 counts. This could reflect increased virus replication following CD4 depletion or vice versa high or prolonged virus replication could cause CD4 depletion via activation and apoptosis, or via release of cytokines that induce lymphocyte marginalization by adhering to endothelial cells [48]–[49]. Given the imbalance between CD4 and CD8 T cells we favor the hypothesis that high or continued viral replication promotes CD4 depletion. Reduced thymic T cell output has been implicated in lymphopenia following H5N1 infection in mice, and is a consequence of infection of dendritic cells that migrate to the thymus and facilitate infection and destruction of thymic cells [50]. It is plausible that thymic output could be compromised in severe 2009 H1N1 because virus has been detected in dendritic cells and thymic epithelial cells from severe but not mild cases; albeit to a far lesser extent and the thymus is not damaged [50]. In this regard, viremia was observed in a severe case but no mild cases and other studies find that viremia is associated with severity and lymphopenia [45], [51]. Reactive hemophagocytosis is seen in lymph nodes and bone marrow of some severe 2009 H1N1 patients [45] and this may contribute to lymphopenia.

Studies of NK cells during human influenza are more limited. One study found that the percentage of NK cells amongst peripheral blood mononuclear cells was low in 3 severe pandemic H1N1 patients with pre-existing conditions compared to 4 mild patients, but the severe patients were not examined until more than 18 days post onset. Another found that NK cells were decreased as a percentage of lymphocytes in children with moderate and severe influenza [52]. NK cells can be directly infected by influenza virus and thereafter killed via apoptosis [53], and this may contribute to NK cell depletion. The present study demonstrating a dramatic reduction in absolute NK and T cell counts in the first few days of symptoms followed by a gradual recovery lends support to the notion that severe illness may be a consequence of impaired NK and T cell responses. Furthermore, T cell and NK cell counts returned to normal following recovery indicating that there is no inherent deficiency.

The loss of both T and NK cells during severe influenza may be linked to the feedback between these subsets required to control influenza virus replication. NK cells play a vital role in the early control of influenza virus replication and their depletion leads to increased morbidity and mortality in animal models [54]–[55]. NK cells bind to and kill infected cells via NK receptor-NKp46 recognition of influenza haemagglutinin [55]. NK cells also promote T cell recruitment by producing IFN-γ, which induces expression of T cell chemoattractants, MIG and IP-10 [56]. In turn, the NK cell response to influenza is enhanced by IL-2 produced by preexisting influenza-specific memory T cells [57], which helps to counteract killing or inhibition of NK cells by influenza virus [53], [58]. Thus, NK cell responses may be overwhelmed if memory T cells are lacking and high or prolonged virus replication may subsequently lead to CD4 T cell depletion via the mechanisms described earlier. In this and other reported studies it has been difficult to determine if severity is associated with differences in virus replication because of late presentation of severe cases [41], [45], however there is some evidence of a slower decline in virus shedding in severe cases [45]. It is also possible that T cells accumulate in the lungs of severe influenza patients and contribute to pathology. However, lung tissues from infants with fatal influenza demonstrate a near absence of CD8 T cells and NK cells such that severity has been attributed to inadequate rather than excessive immune responses [59].

Mild influenza illness was associated with transient expression of activation markers CD38 and HLADR on a small percentage and number of CD8 T-cells. Most HLADR+CD38+ cells will be influenza specific [29] with a minor fraction specific for persistent viruses [60]. As found elsewhere, activated cells could be detected within 3 days of mild influenza onset [60] consistent with the time required for memory cells to respond [61]. CD8 T cell activation was accompanied by depletion of CD8 T-cells with a resting (CD27+CD28+) phenotype without accumulation of partially (CD27−CD28−) or fully (CD27−CD28−) differentiated phenotypes indicating that differentiated effectors migrate to the site of infection. Cells expressing CXCR3, a chemokine receptor expressed by activated T cells that facilitates extravasation into inflamed sites [62], were also depleted. CD8 T cell activation and differentiation varied markedly within the severe patient group. In some activation was excessive and resting phenotype cells were depleted with concomitant accumulation of partially differentiated cells. In most of the remaining severe patients activation was delayed, peaking more than 9 days post onset when activation was resolving in mild illness. A similar phenomenon has been described for CD4 T cells whereby severe infection is associated with the production of anergic rather than effector CD4 T cells [41]. Studies of HIV indicate that partially differentiated CD8 T cells may have limited antiviral function because they fail to produce perforin and TNF-α and accumulation is associated with symptom progression [31], [63]. Aberrant or delayed CD8 activation and differentiation may also affect recruitment to the lungs where they are required to support NK cells and control virus replication.

This study represents a comprehensive analysis of a relatively large group of clinically well-characterized 2009 H1N1 patients but has a number of limitations. The late presentation of severe patients is such that we can not exclude the possibility that lymphocyte activation occurred before presentation. For the same reason viral loads were not informative. We rarely detected viral RNA in respiratory specimens collected from severe patients after enrolment, particularly using the quantitative PCR, which was less sensitive than the CDC influenza A real-time PCR. Although respiratory samples collected during screening were all positive for 2009 H1N1 in the PCR, they were not assessed in the quantitative PCR because nose and throat swabs were pooled for screening but collected separately following enrollment. Commencement of oseltamivir treatment was also relatively delayed in severe patients and this may have contributed to severity, although virus clearance times did not differ significantly. It was not possible to obtain sufficient blood to determine the frequency of influenza specific cells by ex-vivo restimulation of purified mononuclear cells so we could not verify whether HLA-DR+ CD38+ are influenza specific. Finally, the ability to assess effector cells is limited because they appear to be very transient within the blood compartment.

Despite the limitations the results demonstrate that severe influenza is associated with transient T and NK cell deficiency and with delayed or aberrant CD8 effector cell development. Importantly, the patients studied had no known pre-existing illness or obvious inherent NK or T cell deficiency yet succumbed to a virus that generally causes little or no illness. In contrast to NK cells, T cell responses depend on past infection history and can therefore vary widely between healthy individuals. CD8 responses could be delayed or aberrant during severe influenza because pre-existing memory T cells are lacking. Longitudinal cohort studies are required to verify this and the role of memory T cells in clinical protection against influenza.

## Materials and Methods

### Patient recruitment and clinical investigations

This study was conducted at the National Hospital of Tropical Diseases (NHTD), a 160 bed tertiary care centre for adult patients with infectious disease in Ha Noi that also serves as a referral centre for general tropical disease in northern Viet Nam. All patients presenting with influenza-like-illness (ILI) during the first wave of the 2009 H1N1 pandemic were tested by RT-PCR (as described below) and admitted if positive, in accordance with hospital and Ministry of Health policy at the time. ILI was defined as a history of fever within 7 days prior to presentation plus any two of cough, myalgia, lethargy, sore throat or runny nose.

Patients admitted with virologically confirmed influenza were asked to participate in oseltamivir treatment trials (NCT00298233 [standard vs double dose oseltamivir in severe influenza] and NCT00985582 [oseltamivir treatment of 2009 H1N1]). The immunological investigations described in this study were performed as part of these trials. Trial protocols were approved by the NHTD scientific and ethical committee and by the Oxford University Tropical Research Ethics Committee. Patients or a parent or legal guardian provided written informed consent to participate. In NCT00298233 patients aged over 1 year with severe virologically confirmed influenza with illness for <10 days were enrolled into an oseltamivir dosage trial and randomized to standard or double dose. Severe illness was defined as one of the following: new infiltrate on chest X-ray; severe tachypnea (respiratory rate ≥30 for age's ≥12 years); severe dyspnoea (unable to speak full sentences or use accessory respiratory muscles); arterial oxygen saturation ≤92% on room air by trans-cutaneous method; requiring mechanical ventilation at presentation. Patients were excluded from enrollment if they had received more than 72 hours of oseltamivir (six doses) or received oseltamivir at higher than standard doses within the last 14 days. In NCT00985582 patients aged over 1 month with virologically confirmed 2009 H1N1 influenza were enrolled into a single arm clinical, virological and pharmacological study to assess the use of oral oseltamivir. Mild influenza was defined as fever within the past 7 days plus any two of cough, myalgia, lethargy, sore throat or runny nose. Only patients aged >15 years with RT-PCR confirmed 2009 H1N1 and without underlying conditions were analysed in this study.

Patients were examined daily during hospitalization by a team of physicians with experience in influenza diagnosis and treatment. Chest X-ray was performed at enrollment, day 5 and 10.

### Virology

Nasal and oropharangeal (throat) swabs were collected at screening, and on study days 0–10 and 14 using MicroTestM4® viral collection kits (Remel, Lenexa (KS), USA). If the patient was mechanically ventilated an endotracheal aspirate was collected. All respiratory samples were assessed by real-time reverse-transcriptase polymerase chain reaction (RT-PCR), according to WHO/USCDC protocols (CDC reference no. I-007-05, Accessed November 30, 2009, at http://www.who.int/csr/resources/publications/swineflu/CDCRealtimeRTPCR_SwineH1Assay-2009_20090430.pdf). Quantitation of influenza A RNA was performed on positive samples using a real-time RT-PCR assay, as described previously [64]. The PCR uses primers that amplify a 95 bp section of the M gene: forward 3′-GACAAGACCAATCCTGTCACCTCTG-5′, reverse 3′AAGCGTCTACGCTGCAGTCC-5′, probe bp 190 5′TTCACGCTCACCGTGCCCAGTGAGC3′. The limit of detection of this assay is 1000 copies of RNA per milliliter.

### Immune Phenotyping

Two ml venous blood samples were collected into ethylenediaminetetraacetic acid (EDTA) vacutainers on study days 0, 2, 5 and 28 for patients NCT00985582 and on days 0, 5, 10, 14 and 28 for patients in NCT00298233. All monoclonal antibodies and reagents were obtained from BD Biosciences (San Jose (CA), USA). Absolute counts were performed within 8 hours of blood collection using a single-platform, lyse no-wash procedure. Briefly, 50 µl of whole blood was incubated with 10 µl of Multitest 6-color TBNK Reagent in TruCount tubes for 20 minutes. 450 µl of FACSlysing solution was added and cell counts determined within 1 hour on a FACSCanto machine (BD Biosciences) using CD45 versus side scatter to gate on lymphocytes and CD3 to gate on T cells. The activation and differentiation status of T cell subsets was determined by 6-colour flow cytometry. The activation marker mix consisted of HLA-DR (FITC) (2 µl), CD69 phycoerythrin (PE) (2 µl), CD38 PerCP-Cy5.5 (1 µl), CD3PECy7 (2 µl), CD4 APC (2 µl), CD8 APC-Cy7 (1 µl). The differentiation marker mix consisted of CD27 FITC (2 µl), CD28 PE (2 µl), CD3PECy7 (2 µl), CD4 APC (2 µl) and CD8 APC-Cy7 (1 µl). Cells were stained and assessed as above with the exception that forward scatter versus side scatter was used to define the lymphocyte gate. All samples were assessed using the same quadrants to define the percentages of CD8+ and CD4+ CD3 T cells expressing CD27 and CD28 or CD38 and HLA-DR.

### Analysis

Illness day was calculated from the first date of ILI symptoms, which was assigned as day 0. Proportions were compared using odds ratios and Chi-Square or Fishers exact test when any expected cell count was less than 5. Continuous variables were presented as medians and 10–90% ranges and compared using Mann Whitney tests. In patients with mild illness lymphocyte counts and percentages at different days from onset were compared using paired t-Test.

Cut-offs for identifying lymphopenia were derived from values for recovered patients (n = 49) assessed during follow-up visits in this and other studies. Normal ranges, defined as the geometric mean +/−2 standard deviations, were as follows: CD45/lymphocytes 1328–3440; CD3 T cells 900–2653; CD4 T cells 451–1209; CD8 T cells 250–1335; CD19 B cells 82–533; and NK cells 147–558.

Lymphocyte counts and percentages were also correlated with their Pandemic Medical Early Warning Score (PMEWS) score [65], adapted to include 1 point each if the patients was hospitalized ≥14 days or was given supplemental oxygen and 2 points if they were mechanically ventilated. Pearson's correlation was used for normally distributed variables and Spearmans for non-parametric variables, i.e. variables for which the Shapiro Wilks test was significant.

Analyses were performed with SPSS for Windows, Rel. 14.0.0.245, 2005 (SPSS Inc. Chicago (IL), USA).

## Supporting Information

Figure S1 **Patients enrolled and excluded from analysis.**
(TIF)

Figure S2 **Association between PCR cycle threshold (Ct) value for viral RNA detection and CD4 count.** Results are shown for severe (filled circles) and mild (open triangles) patients with a Ct value <40 in CDC influenza A realtime PCR that had peripheral blood CD4 count performed on the same day. Results are shown for illness days 3 (red), 4 (blue), 5 (green), and 6 (purple). Ct values represent the number of cycles required for PCR product levels to exceed the detection threshold and increase as the concentration of viral RNA decreases.
(TIF)

Figure S3 **Representative FACS plots showing activation and differentiation marker expression by CD8 T-cells.** Contour plots depict HLADR versus CD38 and CD27 versus CD28 marker expression by CD3+ CD8+ T-cells from 1 mild influenza patient with ∼ median values for all markers, and 3 severe influenza patients with different expression profiles.
(TIF)
