# Supplementary figures and images for: Severe Pandemic H1N1 2009 Infection Is Associated with Transient NK and T Deficiency and Aberrant CD8 Responses
Source: PLoS One. 2012 Feb 20;7(2):e31535. doi: 10.1371/journal.pone.0031535 (PMC3282732; doi:10.1371/journal.pone.0031535)

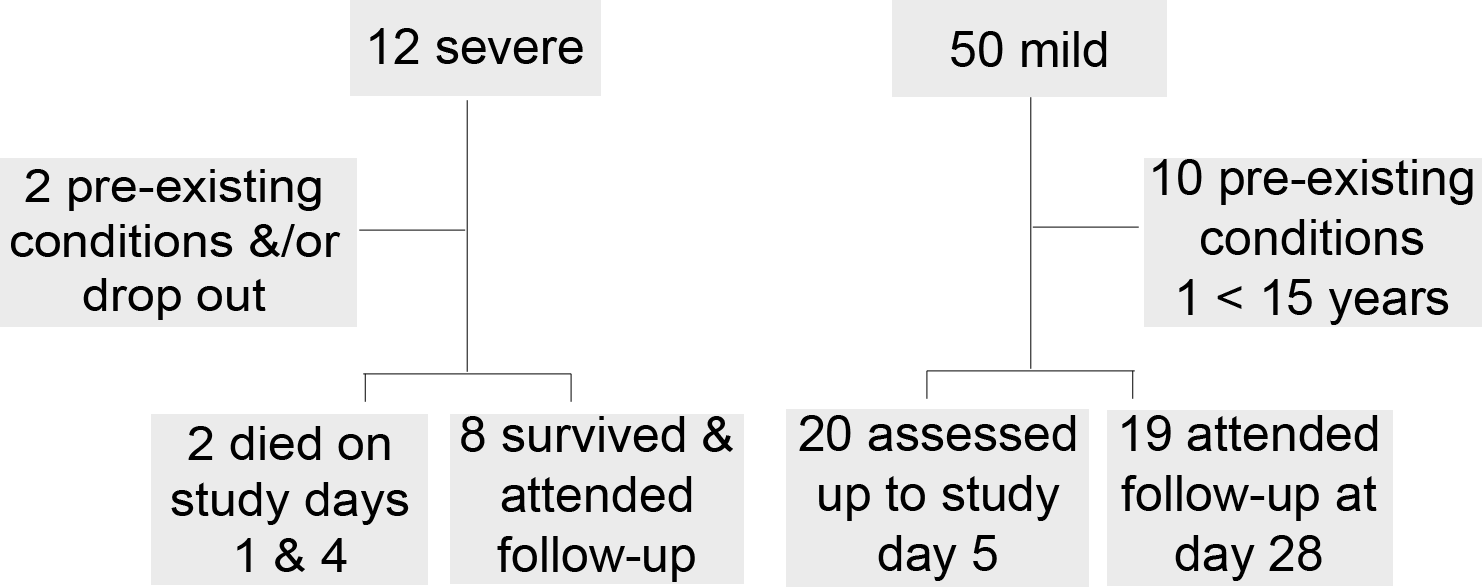

Supplement: Figure S1 — Patients enrolled and excluded from analysis. (TIF) [file pone.0031535.s001.tif]

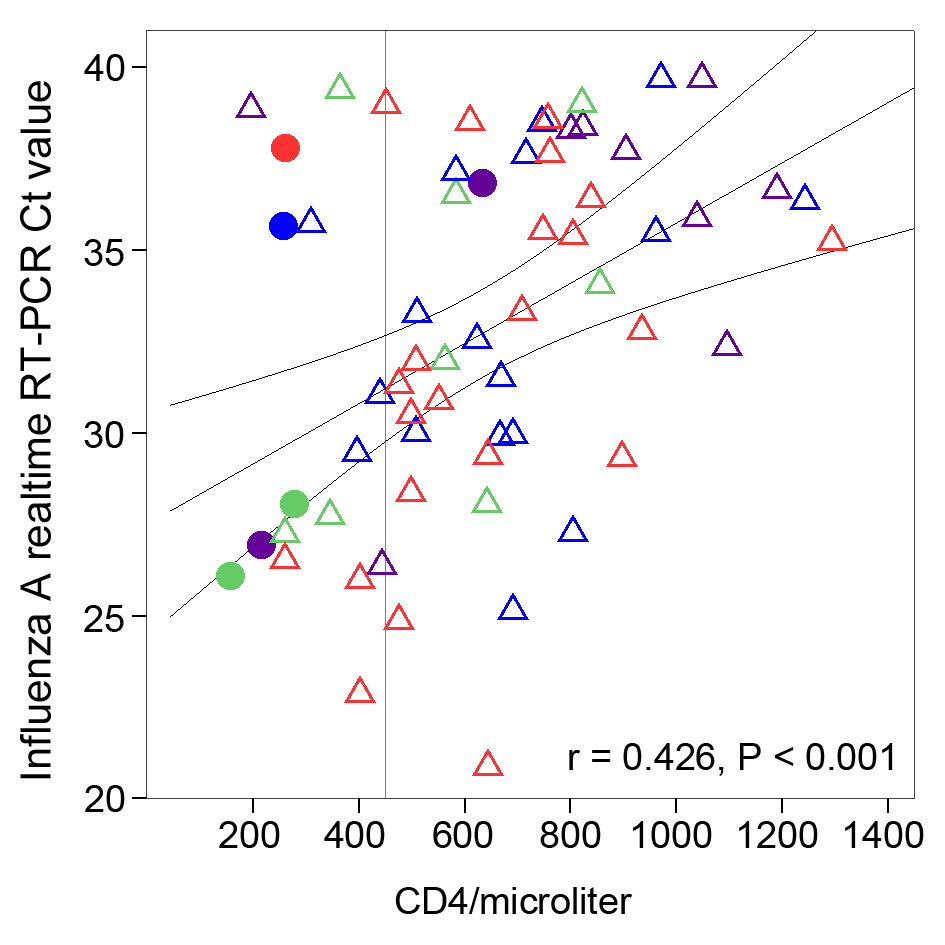

Supplement: Figure S2 — Association between PCR cycle threshold (Ct) value for viral RNA detection and CD4 count. Results are shown for severe (filled circles) and mild (open triangles) patients with a Ct value <40 in CDC influenza A realtime PCR that had peripheral blood CD4 count performed on the same day. Results are shown for illness days 3 (red), 4 (blue), 5 (green), and 6 (purple). Ct values represent the number of cycles required for PCR product levels to exceed the detection threshold and increase as the concentration of viral RNA decreases. (TIF) [file pone.0031535.s002.tif]

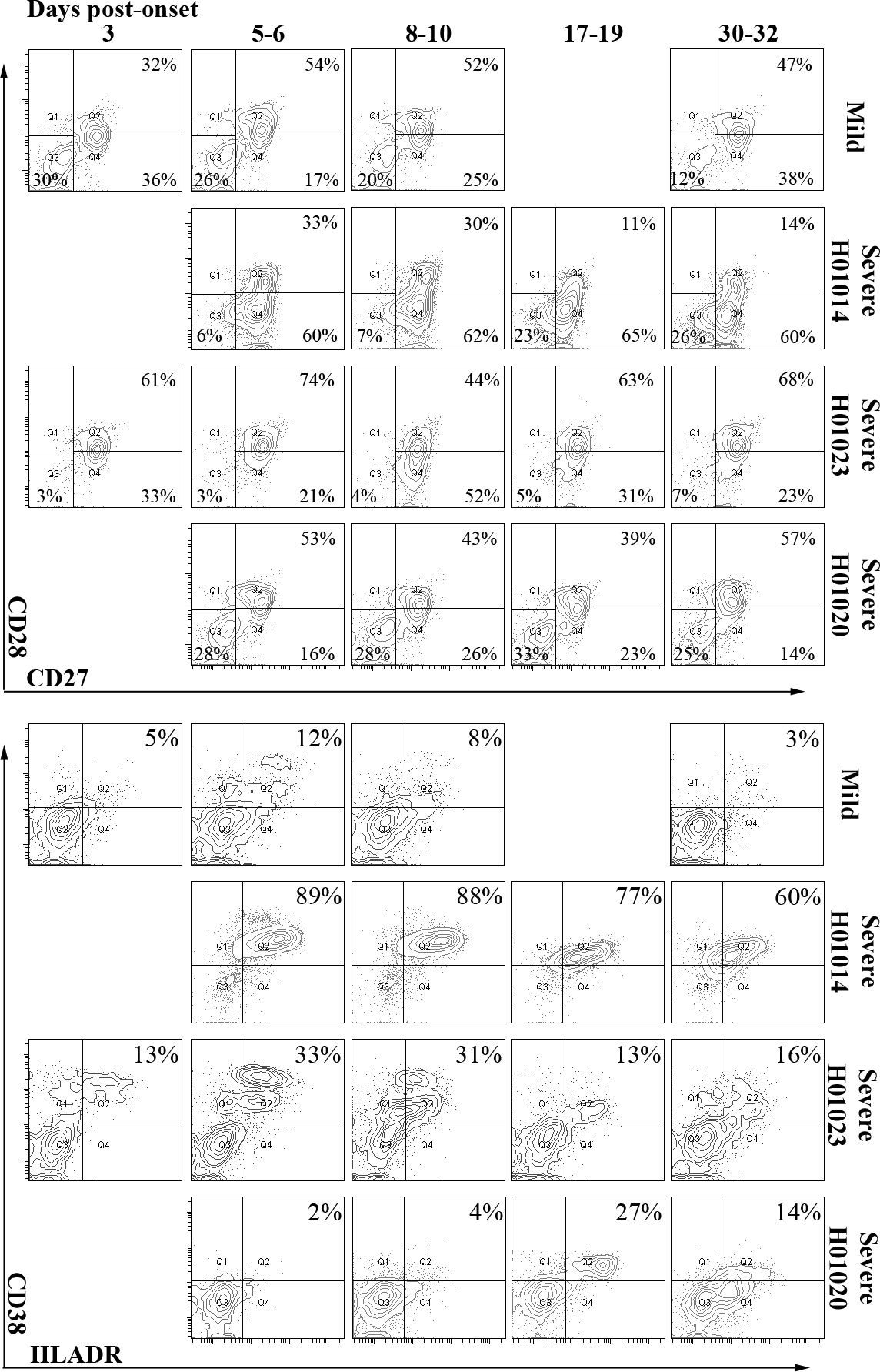

Supplement: Figure S3 — Representative FACS plots showing activation and differentiation marker expression by CD8 T-cells. Contour plots depict HLADR versus CD38 and CD27 versus CD28 marker expression by CD3+ CD8+ T-cells from 1 mild influenza patient with ∼ median values for all markers, and 3 severe influenza patients with different expression profiles. (TIF) [file pone.0031535.s003.tif]
